# Perinatal complications and maximising lamb survival in an adolescent paradigm characterised by premature delivery and low birthweight

**DOI:** 10.1371/journal.pone.0259890

**Published:** 2021-11-15

**Authors:** Jacqueline M. Wallace, Paul O. Shepherd, John S. Milne, Raymond P. Aitken

**Affiliations:** Rowett Institute, University of Aberdeen, Aberdeen, United Kingdom; University of Florida, UNITED STATES

## Abstract

The competition for nutrients in overnourished and still-growing adolescent sheep negatively impacts gestation length, colostrum supply and lamb birthweight, all of which may affect neonatal morbidity and survival to weaning. Herein perinatal complications and the requirement for supplementary feeding were analysed in relation to gestational-intake, and the degree of premature delivery and prenatal growth-restriction exhibited. Pregnancies were established by embryo transfer and the mean/standard deviation (SD) gestation length and birthweight of the optimally-fed control group (n = 100) was used to define early delivery and reduced birthweight categories (1.5 and 3.0 SDs below the control mean for each aspect). Control lambs were largely delivered at term (94%), and had a normal birthweight (92%), while very preterm (≤139days, 18.5%) and preterm delivery (140-142days, 54.8%), extremely low birthweight (ELBW; females ≤2838g and males ≤3216g, 21.1%) and low birthweight (LBW; females 2839 to ≤4001g and males 3217 to ≤4372g, 32.2%), were common in the overnourished group (n = 270, P<0.001). Accordingly, overnourished dams were more likely to lamb without assistance while the incidence of major dystocia was greater in controls. Initial lamb vigour at birth was independent of gestational-intake, delivery or birthweight category but both ELBW and very premature lambs required more assistance with feeding in the first 24h postnatal, primarily reflecting low colostrum availability. Indeed, relative to normal, ELBW lambs had a greater risk of experiencing mismothering, and enhanced likelihood of requiring supplementary feeding throughout the neonatal period (P<0.001). ELBW lambs also had a greater possibility of respiratory issues at birth (P<0.01) and renal complications (P<0.001), while very preterm delivery was associated with an increased risk of gastrointestinal tract problems (P<0.01). In spite of these complications, all-cause mortality was low (5.4%) suggesting that our proactive neonatal care regime can overcome many of the issues associated with extreme prematurity and low birthweight.

## Introduction

Pre-weaning lamb morbidity and mortality limits the reproductive output and profitability of sheep farming and is a serious welfare concern. While rates vary considerably amongst indoor versus extensive (outdoor) systems, and between experimental and ‘real’ farm enterprises, a persistently high average mortality rate of 15% is documented in publications spanning the last 40 year period [1]. The majority of deaths occur in the first three days postpartum and reflect both parturition-related and neonatal losses [2–4]. The former primarily include stillbirths, dystocia, birth injuries, and starvation-mismothering while low birthweight, litter size, male sex and primiparity are well-established risk factors for neonatal mortality [1, 5–10]. Premature birth is also likely to contribute to perinatal complications and mortality in sheep, as it does in humans [11], but there is a paucity of data due to a lack of on farm recording of precise mating dates. The limited information available for well managed flocks (n = 1077 pregnancies) following spontaneous parturition [12] suggests that preterm delivery equivalent to a 2.5% to 5% reduction in gestation length (140–143 days) is associated with an 15% increase in mortality above that of lambs delivered at term (10%, 144–150 days). In the small number of lambs delivered at 137–139 days, mortality was 90% indicating that sheep do not tolerate early delivery well.

One potential approach to improving the lifetime reproductive performance of female sheep is to breed them for the first time during adolescent life (7–9 months of age), but uptake of this management technique is universally low [13]. This reflects a plethora of issues associated with establishing and maintaining pregnancy in adolescent females, and successfully rearing the resulting offspring to weaning. Our overnourished adolescent sheep model was originally developed as an exemplar for studying the competition for nutrients and adverse pregnancy outcomes that occur when gestation coincides with incomplete or continuing maternal growth in very young human adolescents [reviewed in 14], but it also offers insight into issues relevant to ruminant reproduction *per se*. To this end, we establish a singleton pregnancy via assisted conception procedures thereby bypassing some of the problems associated with trying to breed adolescent sheep naturally, including a variable onset of puberty, a transient initial breeding season, failure to be mated, poor quality embryos and high embryo loss [13, 15, 16]. Relative to optimally nourished control adolescents of equivalent age, those who are overnourished exhibit high gestational weight gains and fat accrual at the expense of the conceptus. Placental development and the fetal nutrient supply chain are compromised and premature delivery of low birthweight lambs ensues [17–20]. Furthermore, colostrum supply immediately after parturition is attenuated and together these unfavourable pregnancy outcomes form a perfect storm for increased lamb morbidity and mortality. Indeed lamb mortality was unacceptably high (62%) in the initial studies describing this model [17] necessitating the development of a proactive neonatal care regime involving the ready availability of obstetrical assistance, frequent monitoring of lamb wellbeing, and a high degree of human intervention to ensure adequate nutrition.

The objective of this retrospective analysis of a large cohort of identically managed adolescent pregnancies was to quantify maternal dystocia, lamb vigour, maternal behaviour and the requirement for- and consequences of- supplementary feeding during the neonatal period on morbidity and mortality, and growth to weaning. The primary hypothesis was that the risk of perinatal complications and mortality would be greatest in the most prenatally growth perturbed and/or premature lambs. We also postulated that growth to weaning would reflect the perinatal experiences of the lamb.

Accordingly, we demonstrate that the smallest and most premature lambs have an easy extra uterine passage and are initially vigorous, but in line with our hypothesis they are more vulnerable to mismothering, starvation, and morbidity. The proactive approach to neonatal care described herein limits the impact of these perinatal complications and 95% of young adolescent ewes successfully rear a lamb to weaning. These findings are germane to animal scientists, livestock managers and veterinary practitioners concerned with maximising lamb survival across settings.

## Methods

### Pregnancy establishment and associated husbandry

Procedures were licensed under the UK Animals (Scientific Procedures) Act of 1986 and authorised locally by the Rowett Institute’s Ethical Review Committee. Animals were housed under natural lighting conditions in individual open-wide bar pens that allowed nose-to-nose interactions with neighbouring animals. Pregnancies were established by assisted conception procedures exactly as described earlier [21]. In short, adult ewes (Border Leicester x Scottish Blackface) in prime breeding condition (adiposity score 2.25 or 2.5 units), and of known reproductive history (third or fourth parity) were superovulated and intrauterine inseminated to act as embryo donors. On day 4 after insemination embryos were recovered and grade 1 morula, appropriate for stage, were synchronously transferred into the hormonally-primed uteri of adolescent recipients (Dorset Horn x Greyface) to create singleton pregnancies. Adults were preferentially used as embryo donors since embryos from adolescent ewes have an innately low viability when transferred into either an adolescent or adult uterus [22, 23]. The adolescent recipients were selected from the Rowett Institute’s flock which was free from Enzootic abortion. As lambs they received two doses of a 7 in 1 Clostridial plus Pasteurella vaccine 6 weeks apart (Heptavac P Plus; Intervet UK Ltd., Milton Keynes, UK). They were also vaccinated against Toxoplasmosis ~ 6 weeks before breeding (Toxovax; Intervet UK Ltd.) and sheared ~ 2 weeks prior to embryo transfer to avoid the stress of doing so once pregnancy was established. Embryos were transferred on 35 separate days in 6 different years during the first half of the natural breeding season (mid-Nov to late-Dec) when the adolescents were ~7.5 months old, peripubertal, and had reached similar initial live weight (44.1±0.26kg) and external adiposity score (2.3±0.01). The latter is equivalent to 23% body fat, based on a subjective 5-point body condition scale verified against chemical fat percentage of the fleece free empty body [24]. Body weight at conception was equivalent to approximately 70% of the mature body weight of primiparous ewes of the same genotype at 20 months of age [25]. Viable pregnancies were confirmed by transabdominal ultrasound at ~ day 45 and again at ~ day 85 of gestation.

### Nutritional management during pregnancy and lactation

Commencing directly after embryo transfer, adolescent recipients were offered either a control (n = 128) or high (n = 381) level of a complete diet providing 12 MJ of metabolisable energy (ME) and 140 g of crude protein per kg—composition as detailed previously [26]. The diet was prepared as required on site and fresh food was offered twice daily at 08:00h and 16:00h. In the optimally-fed control group the dietary level was calculated to preserve the original maternal adiposity level throughout pregnancy and to provide 100% of the estimated metabolisable energy and protein requirements of the adolescent sheep carrying a single fetus (normal fetoplacental growth) [27]. Practically this is achieved via a target gestational weight gain of 75g/day for the first two thirds of gestation followed by individual weekly stepwise increases in rations to meet the evolving nutrient needs of the fetus during the final third of pregnancy. This permits a small degree of maternal growth over the course of the entire pregnancy. In contrast, the high ration was fed *ad libitum* throughout pregnancy to support continued maternal growth and increasing adiposity at the expense of the conceptus: to achieve this, recipients had the ration increased stepwise over a 2-week period until the daily food refusal was ~15% of the amount offered. The individual ration offered was reviewed three times weekly to maintain the aforementioned level of food refusal—these animals were deemed overnourished (~2.25 x control intake). To ensure these nutritional targets were achieved weight was recorded fortnightly during the first two thirds of gestation while external adiposity scores were initially measured monthly during the first two thirds, then fortnightly during the final third of pregnancy (same individual in all cases). After parturition all ewes were offered the same complete diet to appetite (*ad libitum*) to maximize milk availability. For the control group this was achieved step-wise over a period of approximately 10 days.

### Perinatal management, interventions and data recorded

All adolescent dams received a further vaccination with Heptovac P Plus at ~ 100 days gestation. This was to boost antibody production and provide passive immunization against clostridial diseases (via ingestion of colostrum) to their lambs. Straw bedding on top of a layer of sawdust was used from ~day 130 of gestation, and this was removed and replaced every 48h. As overnourished adolescent dams consistently deliver early, ewes were supervised 24h per day during the expected delivery period beginning at day 135 of gestation and for a further 72h after the last control birth on day 150. Importantly this involved the same three experienced individuals (RPA, JSM, JMW) working overlapping shifts to ensure a consistent approach to perinatal care and record keeping. The fourth member of the team blinded to nutritional treatment (PS) retrospectively summarized the individual records and categorised the data.

Ewes were allowed to spontaneously labour and lambing assistance matched requirement and was recorded in order to assign a dystocia category (n = 5). The latter ranged from no assistance through minimal, moderate and major assistance to surgical delivery. The requirement for surgical delivery via Caesarean Section was low and was due to either fetal oversize, or a failure of the cervix to dilate appropriately following administration of a uterine relaxant / musculotropic stimulator (Monzaldone, 200mg Vetrabutine hydrochloride i.m., administered twice in 30 minutes, Boehringer Ingelheim Ltd.) to treat Ringwomb. After delivery any initial breathing issues were noted. A suction-pump was used to remove mucous from the mouth, nose and trachea followed by administration of a respiratory stimulant if warranted. The latter involved sublingual application of 5 drops of Dopram V (Doxapram Hydrochloride, 20mg/ml, Zoetis UK Ltd.), and if no immediate response, was followed by administration of Millophylline V (140mg Etamiphylline camsylate i.m., Arnolds). A small number of lambs with obvious cyanosis additionally received supportive oxygen therapy for up to 12h after birth. Ewes were allowed to smell and lick the lamb before it was dried, weighed and girth at the umbilicus measured. The towel used to dry the lamb was left in the pen to aid olfactory recognition. Once delivered the placenta was dissected and its component parts weighed. A standardized proactive regimen of neonatal care was applied to all lambs irrespective of the degree of prematurity or extent of prenatal growth restriction. This involved administration of a vitamin E and selenium supplement (Vitesel, 0.5ml i.m., Norbrook Laboratories) at birth, a 5-day prophylactic course of antibiotics (10% Baytril, 2.5mg enrofloxacin/kg bodyweight, s.c., Bayer Corp.), frequent weight recording and supplementary feeding if required. To further reduce the risk of infection the lamb’s navel was dipped in iodine at birth and at 12h of age. Oxytocin (10iu, Intervet UK Ltd.) was administered to dams i.v. to induce milk let-down and the udder stripped by hand to determine the initial colostrum yield. The colostrum was weighed and in most cases sampled before being fed back to the lamb. Bottle-feeding was considered the optimal feeding route to preserve the strong innate suckling ability normally present in vigorous lambs at birth. If the bottle was refused, less vigorous lambs received part or all of this initial feed via a stomach tube but this was the route of last resort (all recorded). All feeding equipment was continuously cycled through a proprietary baby steam steriliser. Birthweight determined the volume and timing of the initial colostrum feed: due to their small stomach size and potentially immature kidneys, lambs ≤2500g received an initial colostrum volume of 100ml split evenly between birth and 2hours later, while lambs >2500g received 50ml per kg bodyweight at birth. Where initial colostrum yield was less than the 50ml per kg birthweight required to avoid hypothermia [28], colostrum from a frozen donor pool was used to supplement. Viability checks and weight recording were carried out at 4h intervals for a minimum of 72h after birth but continued for as long as required. If lambs were awakened from sleeping, they were observed to see if they stretched on standing and showed immediate interest in suckling their dams—both behaviours indicated wellbeing. Any urination or defaecation at this point was also noted. Any lamb that failed to spontaneously suckle and/or gain weight over an 8h period was supplemented with colostrum (first 24h), ewe milk (harvested from own mother or donor ewe), or formula milk for as long as deemed necessary, and the number of feeds recorded. Every effort was made to avoid the use of formula milk in premature and/or extremely low birthweight lambs as we had prior experience that this was not well tolerated by the immature gut and/or kidneys. The route of supplementary feeding was also recorded and combined with the number of feeds to provide a four-category index of the degree of assistance or lamb vigour during the first 24h. This ranged from: (i) No assistance beyond the initial feed; (ii) Minimal assistance involving one or more feeds by bottle; (iii) Moderate assistance involving one or two feeds by stomach tube; (iv) Major assistance involving three or more feeds being administered solely by feeding tube. Typically, lambs were weighed and observed 6 times in the first 24h after birth and were considered to have received major nutritional supplementation if they required 4–6 feeds by bottle or tube during that time. The upper limit on the volume of colostrum administered during this period was 200 ml per kg birthweight. In some instances the requirement for supplementary feeding extended beyond, or first became apparent after 24h and in this instance 4 or > feeds by tube or bottle from 24 to 96h was used to define marked nutritional supplementation. In all cases, the volume of milk offered was appropriate for the current weight of the lamb.

The neonatal feeding records additionally included notes on maternal behaviour (or mismothering) and these allowed three categories to be defined: (i) An appropriate ewe-lamb bond when the ewe actively encouraged the lamb to seek the udder and stood still to allow it to suckle freely; (ii) An intermediate category where the ewe appeared nervous of the lamb, moving and backing away from it and/ or the normal milk let-down response appeared inhibited requiring the ewe to be gently restrained to allow the lamb to latch-on and suckle; (iii) Inappropriate aggressive behaviour where the ewe attempted to kick or box the lamb repeatedly when it tried to suckle or the lamb was actually harmed. The latter typically involved breaking a leg or ribs hence if the ewe was observed persistently attempting to harm, the lamb was pre-emptively placed within a protective corner of the pen while allowing the ewe to smell and see it. In these instances, lambs were given the opportunity to suckle under supervision every 4h until an appropriate ewe-lamb bond was formed. The occurrence of these maternal behaviours was quantified during the first 24h postnatal and between 24 and 96h of age.

Individual lambs with additional health issues were identified by observation and/or a failure to thrive/gain weight and treated on an individual basis following clinical examination by the Institute’s Named Veterinarian. The primary morbidity issues recorded were pneumonia requiring antibiotic, anti-inflammatory and/or steroid treatments; gastrointestinal tract infections that did not resolve within 24h of oral rehydration or oral anti-diarrheal treatment, and hence required antibiotics; suspected or confirmed renal issues as evidenced by infrequent, low volume or absent urination and blood/protein in the urine sample where obtained. The latter typically first presents at ~36h of age and is unique to small and premature lambs. It was resolved by replacing ewe milk with an oral electrolyte-energy preparation on two or more occasions up to 24h (Energaid, Norbrook Laboratories)—offered by bottle. A composite neonatal morbidity index was defined when a lamb had one or more complications requiring remedial treatment for pneumonia, a gastrointestinal tract infection or renal issues. Mortality rate includes lambs that died or had to be euthanized for specific welfare reasons within the first 4 weeks postnatal. Maternal health issues after delivery were rare and again treated on an individual basis following veterinary consultation. Lambs were weighed at weaning at 11 weeks of age.

### Definition of preterm delivery and birthweight categories

The extent of preterm delivery and reduced birthweight was categorised using the mean and standard deviation (SD) for optimal control pregnancies. Gestation length was independent of sex and was 145.2±1.89 days for the control group (mean ± SD). Pregnancies were classified as preterm or very preterm delivery if gestation length was less than 1.5 or 3 SD’s below the mean control gestation length, respectively, i.e. 140-142days for preterm and ≤139days for very preterm. Control birthweight was not impacted by year of study (P = 0.333) but as males were heavier than females (Mean ± SD: 5529 ± 771g vs. 5166 ± 776g, P = 0.005) birthweight was defined on a sex-specific basis. Lambs were categorised as low (LBW) or extremely low birthweight (ELBW) when weight at delivery was less than 1.5 or 3.0 SD’s below the sex-specific birthweight of controls. Thus for LBW and ELBW categories: females ≥2839 to 4001g, and ≤2838g, respectively, and for males ≥3217 to 4372g, and ≤3216g, respectively. For the majority of the analysis the remaining lambs were deemed normal birthweight. The exception was in Table 1 where lambs were additionally classified as oversized if birthweight was in the upper quartile for control lambs (5831-7650g). These descriptors are sheep specific.

**Table 1:** Pregnancy outcome including the incidence and extent of premature delivery and prenatal growth restriction in singleton bearing adolescent ewes offered a control intake or overnourished throughout gestation.

Gestational intake	Control	Overnourished	^¢^P-value
Number of pregnancies	100	270	
Gestation length, days	145.2±0.18	141.2±0.13	**<0.001**
Incidence of			
^β^Very preterm delivery, n (%)	0 (0)	50 (18.5)	**<0.001**
^β^Preterm delivery, n (%)	6 (6)	148 (54.8)	**<0.001**
Term delivery, n (%)	94 (94)	72 (26.7)	**<0.001**
Very preterm gestation length, days	n/a	137.9±0.16	n/a
Preterm gestation length, days	141.5±0.34	141.0±0.06	0.204
Term gestation length, days	145.4±0.17	143.7±0.11	**<0.001**
Placental weight, g	429±11.6	311±6.5	**<0.001**
Fetal cotyledon weight, g	141.1±4.32	83.8±2.14	**<0.001**
Lamb girth at umbilicus, mm	39.9±0.32	36.5±0.27	**<0.001**
Lamb birth weight, g	5339±78	4005±67	**<0.001**
Incidence of			
^¥^Extremely low birth weight, n (%)	0 (0)	57 (21.1)	**<0.001**
^¥^Low birth weight, n (%)	8 (8)	87 (32.2)	**<0.001**
Normal birth weight, n (%)	68 (68)	113 (41.9)	**<0.001**
Oversized birth weight, n (%)	24 (24)	13 (4.8)	**<0.001**
Extremely low birth weight, g	n/a	2419±60	n/a
Low birth weight, g	3946±93	3696±37	0.051
Normal birth weight, g	5148±56	4806±42	**<0.001**
Oversized birth weight, g	6346±85	6069±66	**0.034**
Male:female ratio			
Overall	45:55	146:124	0.129
Very premature lambs	n/a	21:29	n/a
Extremely low birth weight lambs	n/a	32:25	n/a
Oversized birth weight	14:10	10:3	0.305

^β^Classified as preterm or very preterm delivery if gestation length was less than 1.5 or 3.0 standard deviations below the mean control gestation length, respectively, i.e. 140–142 days for preterm and ≤139 days for very preterm.

^¥^Classified as low birthweight (LBW) or extremely low birthweight (ELBW) if weight at delivery was less than 1.5 or 3.0 standard deviations below the sex-specific birthweight of the optimally nourished control group. Thus for LBW and ELBW categories: females ≥2839 to 4001g, and ≤2838g, respectively, and for males ≥3217 to 4372g, and ≤3216g, respectively. Lambs classified as oversized if birthweight was in the upper quartile for control lambs (5831-7650g). Continuous data analysed by ANOVA and categorical data by Fisher’s exact test. Significant P values indicated in bold font.

### Data analysis

Data were analysed using Minitab (version19; Minitab Inc., State College, PA). Normality was confirmed and there was no evidence of outliers for pregnancy outcome or neonatal parameters using Grubbs’ testing at 5% significance. Exceptionally the number of supplementary feeds required to ensure survival was not normally distributed and were log transformed. Continuous data were analysed by ANOVA and where this involved three or more groups *post hoc* comparisons used Fishers LSD method at 1%. Categorical data were analysed by either Fishers Exact Test or Chi Squared where appropriate. The risk of neonatal morbidity or mortality, an impaired ewe-lamb bond and requirement for supplementary feeding in relation to delivery and birthweight category was assessed by logistic regression. Risks are presented as Odds Ratios (OR) with 95% confidence intervals (CI) and were adjusted for gestational intake group and lamb sex. Logistic regression was also used to examine the risk of morbidity and mortality in relation to lamb vigour at birth with additional adjustment for gestation length and birthweight as continuous variables. Multiple regression was used to interrogate the relationship between gestation length, birthweight, colostrum yield, sex, maternal dystocia, initial lamb vigour and supplementary milk feeding during the neonatal period on absolute and fractional growth for the population as a whole, and in the extremely low birthweight group. Best sets regression helped identify which aspects could be reasonably omitted to keep the models simple and the predictors retained are detailed in the results text.

## Results

### Reproductive wastage

Conception rate following embryo transfer was 78.9 and 71.4% in control and overnourished dams, respectively (P = 0.106), and there were three late abortions of an autolysed fetus in the final third of pregnancy (1 control and 2 overnourished).

### Gestational dietary intake, pregnancy outcome and dystocia

**Fig 1:**
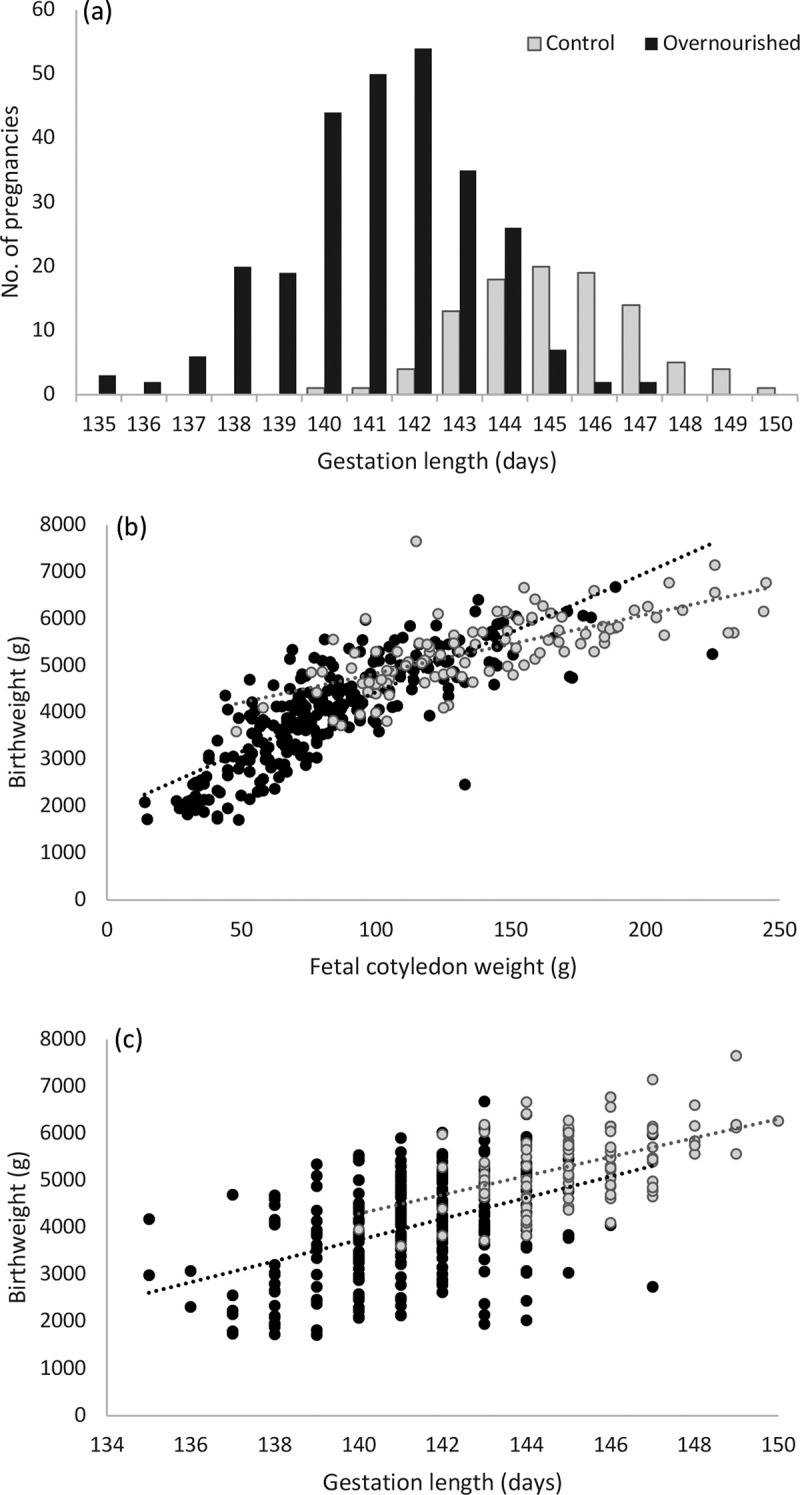
Individual birth parameters for study population in relation to gestational intake. Distribution of gestation length (a) and relationship between total fetal cotyledon weight and birthweight (b), and between gestation length and birthweight (c), in singleton bearing adolescent ewes offered a control intake or overnourished throughout pregnancy. For control (grey symbols) and overnourished (black symbols) pregnancies in (b) r = 0.687 and 0.808, and in (c) r = 0.484 and 0.430, all P<0.001.

By design the fetuses of adolescent ewes receiving a control intake to maintain maternal adiposity throughout gestation had a largely optimal prenatal growth trajectory, gestation length and birthweight (Table 1). Ninety four percent of control lambs spontaneously delivered at term and 92% of lambs had a normal or higher birthweight using the definitions detailed in the methods section. There were no very preterm deliveries or extremely low birthweight lambs in the control group. By contrast, average gestation length was four days shorter in the overnourished pregnancies (Table 1) reflecting a very high incidence of preterm (54.8%) and very preterm delivery (18.5%), with viable lambs born at day 135 of gestation forwards. In addition, average placental mass and fetal cotyledon weight were attenuated in the overnourished group and the incidence of low and extremely low birthweight was 32.2 and 21.1%, respectively. At the other end of the birthweight spectrum 4% of lambs born to overnourished dams had weights at delivery that were in the upper quartile for controls. The distribution in gestation length and the associations between placental weight, gestation length and birthweight by nutritional treatment are shown in Fig 1.

Placental weight and birthweight were highly correlated in both control and overnourished pregnancies (Fig 1B) and for the population overall (r = 0.863, P<0.001). While gestation length was also positively associated with birthweight, the relationship was less strong in both nutritional treatment groups (Fig 1C), and for the population overall (r = 0.605, P<0.001). Thus for the 50 lambs spontaneously delivered between 135 and 139 days gestation, 26 were categorised as ELBW (52%), 12 were LBW and 12 of normal birthweight. For those delivered between 140 and 142 days gestation, 22, 55 and 77 were defined as ELBW, LBW and normal, respectively. Conversely 9 of 57 ELBW lambs, and 28 of 95 LBW lambs were delivered at term. The overall male to female sex ratio was independent of nutritional treatment (Table 1), and within the overnourished group there was no significant difference in the number of males versus females delivered very preterm (P = 0.110) or at extremely low birthweights (P = 0.190). Although total numbers are small there were more males than females categorised as oversized in the overnourished group (P = 0.006), but this did not differ from controls (Table 1).

**Fig 2:**
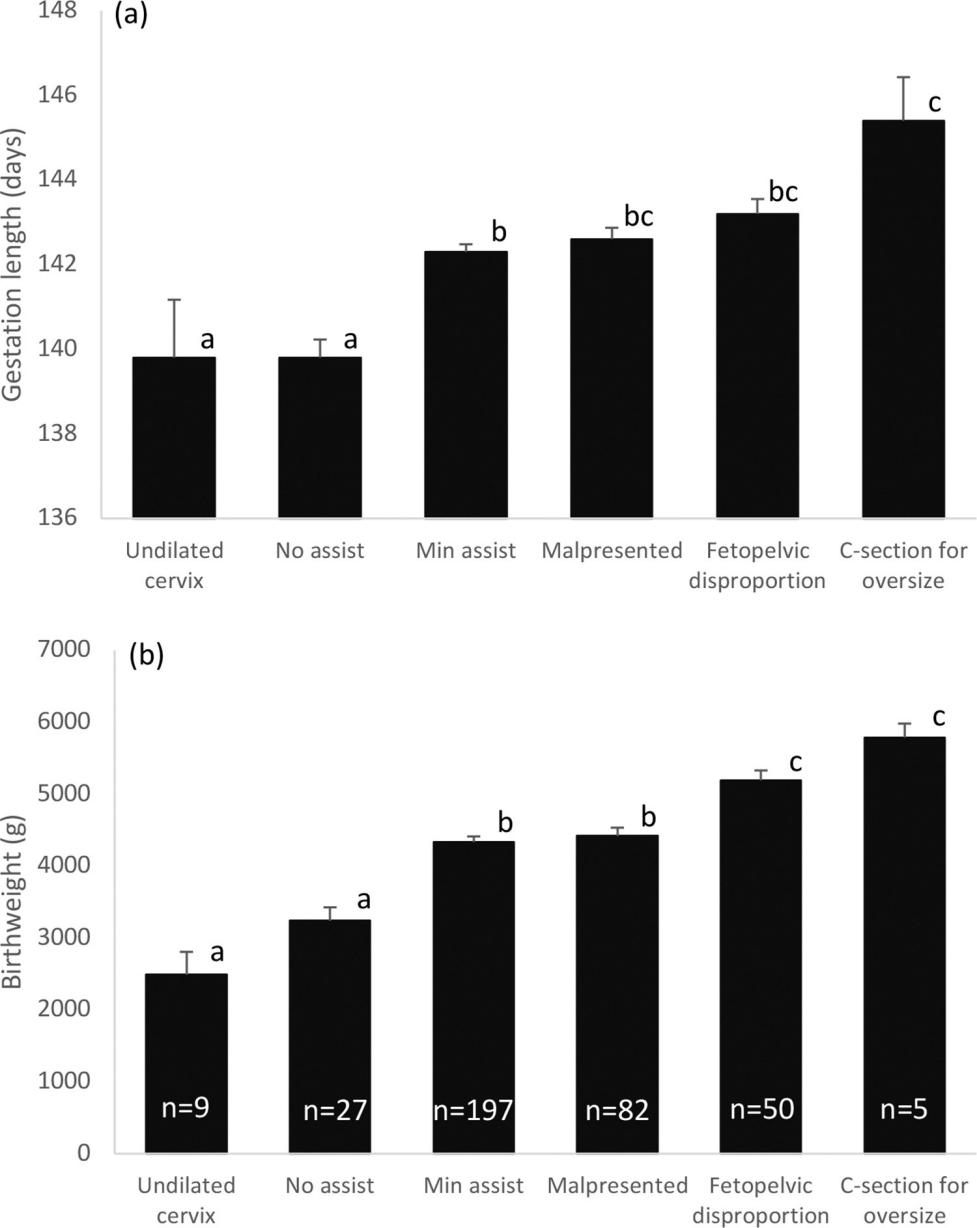
Gestation length and birthweight in relation to dystocia category. Average lamb birthweight (a) and gestation length (b) in relation to degree of assistance required at delivery, and the main underlying cause. Where superscript letters differ P<0.01.

Dystocia categories and their descriptors are shown in Table 2. Sixty percent of lambs were delivered with no or minimal assistance and those that were completely unassisted were exclusively born to overnourished dams. Many of these unassisted deliveries were also unsupervised at the point of birth reflecting both a rapid second stage of labour and low birthweight (Fig 2).

**Table 2:** Incidence of dystocia in singleton bearing adolescent ewes offered a control intake or overnourished throughout gestation.

Dystocia category	Description	Control n (%)	Overnourished n (%)	P-value
No assistance	Unassisted, often unsupervised.	0 (0)	27 (10)	**0.0001**
Minimal assistance	Uncomplicated, ewe could have delivered lamb herself.	56 (56)	141 (52)	0.558
Moderate assistance	Malpresented (e.g. leg(s) back; head back, down or twisted; breach), or fetopelvic disproportion.	12 (12)	51 (18.8)	0.160
Major assistance	Malpresented or fetopelvic disproportion or incomplete dilation of cervix, and requiring considerable sustained effort to deliver lamb. Ewe required pain relief ± antibiotic	29 (29)	47 (17.4)	**0.019**
Caesarean section	Surgical delivery due to fetal oversize or un-dilated cervix.	3 (3)	4 (1.5)	0.393

Conversely, a greater number of controls required a sustained effort to deliver the lamb due to malpresentation and/or fetopelvic disproportion with a degree of difficulty that was judged to require analgesia and/or antibiotic treatment afterwards (Table 2). Irrespective of dietary intake during pregnancy, the level of assistance required to deliver the lamb and the incidence of malpresentation, fetopelvic disproportion and delivery by Caesarean section largely increased with greater gestation length and higher birthweight (Fig 2). The exception was those pregnancies complicated by a failure of the cervix to dilate 6h or more after the fetal membranes appeared at the vulva (Ringwomb, n = 9). All required major assistance (n = 7) or surgical intervention (n = 2) to secure delivery, all were in the overnourished group and all lambs were of low (n = 3) or extremely low birthweight (n = 6), and 7 of 9 were born prematurely. The degree of assistance required was not influenced by lamb sex (overall Chi Square, P = 0.096).

### Colostrum yield, lamb vigour and maternal behaviour

The impact of gestational intake on initial colostrum yield, indices of lamb vigour and maternal behaviour during the first 24h after delivery are detailed in Table 3. In keeping with the known characteristics of this animal model the initial colostrum yield was attenuated by ~60% in overnourished compared with control dams. Consequently, the number of dams with an inadequate volume of colostrum for the weight of their lambs was two-fold higher in the overnourished group and in more than 20% of cases dams had between zero and 50ml colostrum available at parturition, necessitating the widespread use of banked ewe colostrum.

**Table 3:** Initial colostrum yield, lamb vigour and maternal behaviour during the first 24h after delivery in singleton bearing adolescent ewes offered a control intake or overnourished throughout gestation.

Gestational intake	Control	Overnourished	^¢^P-value
Average colostrum yield, ml	467±38.1	186±9.1	**<0.001**
^α^ No. with inadequate colostrum/kg fetus	33 of 97 (34%)	159 of 266 (59.8%)	**<0.001**
No. with less than 50ml colostrum at parturition	3 (3.1%)	58 (21.8%)	**<0.001**
^β^Initial colostrum administered to lamb by			
• bottle (optimum)	83 (86.5%)	232 (87.9%)	0.720
• bottle then stomach tube	10 (10.4%)	19 (7.2%)	0.380
• stomach tube	3 (3.1%)	13 (4.9%)	0.574
^¥^Average no. of assisted feeds per first 24h	0.4±0.08	1.5±0.11	**<0.001**
^β^Lamb vigour category during first 24h			
• no assistance beyond initial feed (optimum)	66 (67.3%)	111 (42.2%)	**<0.001**
• minimal assistance, 1 or > feeds by bottle	15 (15.3%)	111 (42.2%)	**<0.001**
• moderate assistance, 1 or 2 feeds by tube	16 (16.3%)	30 (11.4%)	0.217
• major assistance, 3 or > feeds by tube	1 (1%)	11 (4.1%)	0.192
^β^Maternal behaviour category during first 24h			
• appropriate ewe-lamb bond	92 (93.9%)	243 (92.0%)	0.657
• ewe gently restrained to allow suckling	6 (6.1%)	13 (4.9%)	0.796
• ewe harms or attempts to harm	0	8 (3.0%)	0.114

Values are mean±sem or number (percent). Missing or incomplete colostrum yield data for 3 control and 4 overnourished dams.

^α^Defined based on requirement of 50ml per kg fetal weight.

^β^See methods text for more detail.

^¥^excludes initial colostrum administration.

^¢^Continuous data analysed by ANOVA and categorical data by Fishers Exact test. Significant P values indicated in bold font.

Nevertheless, gestational intake did not influence initial lamb vigour as assessed by the ability to suck the first colostrum feed from a bottle, and the requirement to use a stomach tube to feed weak lambs in the population as a whole was low (<5%). Thereafter lambs born to overnourished dams did on average require more supplementary feeding during the first 24h postnatal but this largely involved one or more feeds being offered by bottle and there was no difference between gestational intake groups with respect to the requirement for moderate or major assistance via tube feeding. Similarly, gestational intake did not significantly influence maternal behaviour in the first 24h with more than 92% of the total population developing an appropriate ewe-lamb bond.

The extent of prenatal growth restriction and/or prematurity is likely to influence colostrum availability, lamb vigour and maternal behaviour and accordingly these parameters have additionally been examined separately by birthweight and delivery categories, independent of gestational intake (Table 4). This reveals that average colostrum yield broadly mirrors average gestation length and birthweight within both the birthweight and early delivery categories. Pregnancies that resulted in zero or very low colostrum availability at parturition were overwhelmingly in the extremely low birthweight group and 70% of mothers had insufficient colostrum to meet their lamb’s initial requirement. To a lesser extent, zero or low colostrum availability was also a feature of both categories of premature delivery and a higher proportion of these ewes were deemed to have an inadequate supply to meet fetal requirement relative to those delivering at term. Neither birthweight nor delivery category impacted initial lamb vigour at the first feed (bottle versus stomach tube), and vigour was also independent of lamb sex (P = 0.248, Chi squared).

**Table 4:** Initial colostrum yield, lamb vigour and maternal behaviour during the first 24h after delivery in relation to birthweight and delivery categories.

Birthweight or delivery category	ELBW	LBW	Normal	^¢^Birthweight P-value	Very premature	Premature	Term	^¢^Delivery P-value
Total no. of pregnancies	57	95	218		50	154	166	
Gestation length, days	139.9±0.32^a^	141.4±0.20^b^	143.3±0.17^c^	**<0.001**	137.9±0.16^a^	141.1±0.06^b^	144.7±0.12^c^	**<0.001**
Lamb birth weight, g	2419±59^a^	3717±36^b^	5157±46^c^	**<0.001**	3123±154^a^	4084±74^b^	5001±80^c^	**<0.001**
Average colostrum yield, ml	95±15.1^a^	179±11.7^a^	339±20.7^b^	**<0.001**	140±19.7^a^	192±12.4^a^	361±25.8^b^	**<0.001**
^α^ No. with inadequate colostrum/kg fetus	39 (70.9)^a^	51 (54.2)^b^	102(49.7)^b^	**0.020**	32 (66.6)^a^	91 (59.9)^a^	69 (42.3)^b^	**0.001**
No. with less than 50ml colostrum at parturition	29 (52.7)^a^	10 (10.6)^b^	22 (10.2)^b^	**<0.001**	17 (35.4)^a^	29 (19.1)^b^	15 (9.2)^c^	**<0.001**
^β^Initial colostrum feed via								
• bottle (optimum)	44 (84.6)	82 (86.3)	189 (87.5)	0.766	36 (80)	136 (88.3)	145 (88.4)	0.287
• bottle then tube	5 (9.6)	7 (7.4)	17 (7.9)	0.895	4 (8.9)	13 (8.4)	13 (7.9)	0.973
• stomach tube	3 (5.8)	5 (5.3)	8 (3.7)	0.728	5 (11.1)	5 (3.0)	6 (3.6)	0.064
^¥^Average no. of assisted feeds during 1^st^ 24h	3.7±0.28^a^	1.3 ±0.14^b^	0.6±0.07^c^	**<0.001**	2.4±0.36^a^	1.4±0.13^b^	0.8±0.10^c^	**<0.001**
^β^Lamb vigour during 1^st^ 24h								
• no assistance beyond initial feed (optimum)	7 (13.7)^a^	36 (38.3)^b^	134 (62.0)^c^	**<0.001**	15 (34.1)^a^	65 (42.5)^a^	98 (59.8)^b^	**0.001**
• minimal assistance, 1 or > feeds by bottle	30 (58.8)^a^	42 (44.7)^a^	54 (25.0)^b^	**<0.001**	19 (43.1)^ab^	64 (41.8)^a^	43 (26.2)^b^	**0.007**
• moderate assistance, 1 or 2 feeds by tube	7 (13.7)	14 (14.9)	25 (11.6)	0.704	6 (13.6)	19 (12.4)	21 (12.8)	0.986
• major assistance, 3 or > feeds by tube	7 (13.7)^a^	2 (2.1)^b^	3 (1.4)^b^	**<0.001**	5 (11.4)^a^	5 (3.2)^ab^	2 (1.2)^b^	**0.004**
^β^Maternal behaviour 1st 24h								
• appropriate ewe-lamb bond	44 (84.6)	86 (90.5)	205 (95.3)	**0.030**	41 (91.1)	141 (92.1)	153 (93.3)	0.861
• ewe gently restrained to allow suckling	4 (7.7)	6 (6.3)	9 (4.2)	0.514	1 (2.2)	9 (5.9)	9 (5.5)	0.615
• ewe harms or attempts to harm	4 (7.7)	3 (3.1)	1 (0.5)	**0.010**	3 (6.6)	3 (2.0)	2 (1.2)	0.068

Values are mean ± sem or number (percent). Missing or incomplete colostrum yield data for 7 pregnancies; no initial vigour score for 7 lambs.

who died at or soon after delivery, no vigour score for a further 2 lambs who died within 24h.

^α^Defined based on requirement of 50ml per kg.

fetal weight.

^β^See methods text for more detail.

^¥^excludes initial colostrum administration. ELBW, extremely low birthweight and LBW, low birthweight, very premature and premature delivery as detailed in the methods text.

^¢^For birthweight and delivery categories separately continuous data analysed by ANOVA followed post hoc by Fishers lsd method and categorical data by chi-squared. Where superscripts differ within rows for birthweight or delivery categories, P<0.01. Significant P values indicated in bold font.

However, the average rate of colostrum supplementation during the first 24h was greatest in the extremely low birthweight (ELBW>LBW>Normal) and very premature groups (very premature>premature>term)—this reflected a higher incidence of both minimal and major assistance being required. Maternal behaviour was slightly impacted by birthweight category and although the incidence was low, the mothers of ELBW lambs were more likely to harm or attempt to harm their lambs. The risk of an impaired ewe-lamb bond (mismothering) and likelihood of requiring supplementary feeding during the first 24h, and from 24-96h postnatal was calculated (Table 5). Relative to the normal birthweight group, those that were extremely small had a 4 to 15-fold higher risk of an impaired ewe-lamb bond during the neonatal period. They were also 33 times more likely to require major nutritional assistance during the first 24h of life, and 7 times more likely to require supplementary feeding between 24 and 96h. Similar risks were evident when the ELBW lambs were compared with the LBW group. In contrast, premature delivery categories *per se* did not impart significant risk for either parameter.

**Table 5:** Risk of an impaired ewe-lamb bond and likelihood of requiring supplementary feeding during the neonatal period in relation to delivery and birthweight category.

		^¥^Impaired ewe-lamb bond, birth to 24h after delivery	^¥^Impaired ewe-lamb bond, 24 to 96h after delivery	4–6 feeds by tube or bottle from birth to 24h	4 or > feeds by tube or bottle from 24 to 96h after delivery
No. of affected lambs	Rate (%)	27 (7.4%)	23 (6.5%)	46 (12.8%)	30 (8.4%)
Categorical predictors					
Very premature vs term	OR (95% CI)	0.64 (0.15–2.70)	0.29 (0.06–1.33)	1.96 (0.59–6.48)	1.70 (0.49–5.92)
Premature vs. term	OR (95% CI)	0.93 (0.33–2.58)	0.75 (0.24–2.32)	1.00 (0.35–2.79)	0.90 (0.30–2.66)
Very premature vs prem	OR (95% CI)	0.69 (0.19–2.48)	0.38 (0.10–1.49)	1.96 (0.73–5.28)	1.89 (0.66–5.40)
ELBW vs. normal	OR (95% CI)	**4.98 (1.51–16.4)** ***	**15.99 (4.72–54.07)** ***	**33.99 (11.56–99.9)** ***	**7.78 (2.60–23.22)** ***
LBW vs. normal	OR (95% CI)	2.50 (0.88–7.06)	1.08 (0.24–4.75)	2.30 (0.74–7.15)	1.56 (0.49–4.93)
ELBW vs. LBW	OR (95% CI)	1.99 (0.67–5.85)	**14.79 (3.78–57.80)** ***	**14.73 (5.68–38.22)** ***	**4.97 (1.69–14.61)** **

Odds ratios and 95% confidence limits from binary logistic regression adjusted for gestational intake group and lamb sex

**P<0.01

***P<0.001.

^¥^Impaired ewe-lamb bond when ewe had to be gently restrained to facilitate suckling or when ewe had harmed or attempted to harm the lamb.

### Neonatal morbidity or mortality, and maternal health

The most prevalent morbidity issues related to respiratory problems at birth (5 control vs. 27 overnourished group lambs, P = 0.148), pneumonia (0 control vs. 9 overnourished lambs, P = 0.119), gastrointestinal complications (1 control vs. 12 overnourished lambs, P = 0.199) and renal difficulties (1 control vs. 18 overnourished lambs, P = 0.031). The latter included 9 lambs that had blood and protein in their urine and low urine volume, and 10 lambs that had low or absent observed urine output and were treated with electrolyte fluid replacement before a sample was obtained. In all cases these lambs had received a degree of supplementary feeding during the first 24h postnatal (mean 4.6, range 2–6).

Twenty lambs died during the period of study, and all before 4 weeks of age. This included 4 lambs born with a strong heartbeat but whose lungs failed to inflate (stillbirths), and 6 lambs born to overnourished dams which were harmed by the mother (broken ribs, crushed/sat on or broken leg(s)) and had to be humanely euthanized. A further 6 lambs had neurological issues (possibly due to peri-partum anoxia) and either died within a few hours or had to be euthanized within 12h of birth as they were floppy, unresponsive and unable to stand. There were two cases of pneumonia that failed to respond to treatment, a large control lamb whose ribs fractured during breach delivery, and a lamb with a congenitally deformed rib cage. Accordingly, all-cause neonatal mortality was 4% in control and 5.9% in overnourished groups (P = 0.608) and was independent of lamb sex (P = 0.441). Although the incidence of morbidity and mortality was greater in the overnourished group only the renal associated issues reached statistical significance compared with the control group. To better explore whether premature delivery and/or low birthweight were risk factors for neonatal complications binary logistic regression was used, with adjustment for gestational intake and lamb sex (Table 6). The model would not fit for pneumonia as there were too few cases but these lambs were included in the composite neonatal morbidity index. Relative to normal lambs, ELBW lambs had a three-fold higher risk of experiencing respiratory issues at birth. They also had a massively increased risk of renal complications in the neonatal period, and this was also evident for the extremely low versus low birthweight comparison. In contrast, the risk of gastrointestinal complications related to the degree of prematurity and was 19 times higher in the very preterm versus term comparison, and 10 times higher in the very preterm versus preterm groups. Given these findings, it is not surprising that both very premature delivery and ELBW increased the risk of neonatal morbidity, irrespective of the comparator. Similarly, the risk of mortality within the first month postnatal was 6-fold greater in very premature compared with premature lambs, and 7-fold higher in extremely low versus normal birthweight groups. The incidence of poor vigour at birth as judged by the requirement to administer the first colostrum feed by stomach tube was low (n = 16 cases), but compared to those that suckled vigorously from a bottle was associated with an increased risk of mortality after additionally adjusting for birthweight and gestation length (OR 12.1 [95% CI 2.79–52.83], P = 0.001). However, initial vigour did not influence morbidity (P = 0.166).

**Table 6:** Risk of respiratory issues at birth, neonatal morbidity or mortality in relation to delivery and birthweight category.

		Respiratory issues at birth	Gastrointestinal tract complications	Renal complications	^α^Neonatal morbidity	Mortality
No. of affected lambs	Rate (%)	32 (8.6%)	13 (3.5%)	19 (5.1%)	38 (10.3%)	20 (5.4%)
Categorical predictors						
Very premature vs term	OR (95% CI)	3.16 (0.87–11.49)	**19.77 (2.41–161.74)** **	0.66 (0.13–3.31)	**4.44 (1.31–15.08)** *	4.57 (0.96–21.58)
Premature vs. term	OR (95% CI)	1.64 (0.52–5.13)	1.94 (0.24–15.15)	0.98 (0.21–4.58)	1.76 (0.57–5.43)	0.74 (0.15–3.55)
Very premature vs prem	OR (95% CI)	1.92 (0.73–5.03)	**10.16 (2.54–40.65)** **	0.67 (0.19–2.33)	**2.52 (1.03–6.13)** *	**6.11 (1.64–22.74)** **
ELBW vs. normal	OR (95% CI)	**3.90 (1.36–11.15)** **	0.21 (0.03–1.28)	**255 (10.77–6082)** ***	**6.98 (2.65–18.34)** ***	**7.54 (1.57–36.20)** **
LBW vs. normal	OR (95% CI)	1.56 (0.56–4.31)	0.73 (0.19–2.84)	4.33 (0.17–107.13)	1.27 (0.43–3.69)	3.22 (0.73–14.21)
ELBW vs. LBW	OR (95% CI)	2.49 (0.90–6.92)	0.28 (0.04–1.83)	**58.99 (6.21–560)** ***	**5.47 (2.05–14.60)** ***	2.33 (0.66–8.19)

^α^ Neonatal morbidity if lamb had one or more complications requiring remedial treatment for pneumonia, a gastrointestinal tract infection which did not resolve within 24h, or renal issues (see methods text for description). Mortality includes lambs that died or were euthanized for welfare reasons within the first 4 weeks postnatal. Adjusted for gestational intake group and lamb sex. Odds ratios and 95% confidence limits from binary logistic regression

*P<0.05

**P<0.01

***P<0.001.

Maternal health issues requiring remedial treatment during the first month postnatal included 3 cases of retained placenta (all overnourished dams, P = 0.566), attempted or actual uterine prolapse (4 control vs. 7 overnourished, P = 0.497), mastitis (2 control vs. 1 overnourished, P = 0.178) and pneumonia (3 controls vs. 12 overnourished, = 0.767). There were no maternal deaths. In addition, 5 overnourished dams failed to establish an appropriate lactation by three weeks post-partum and their lambs (4 extremely low and 1 normal birthweight) were subsequently bottle-reared on formula. These lambs were excluded from the growth to weaning analysis detailed below.

### Growth in the neonatal period, and from birth to weaning

**Fig 3:**
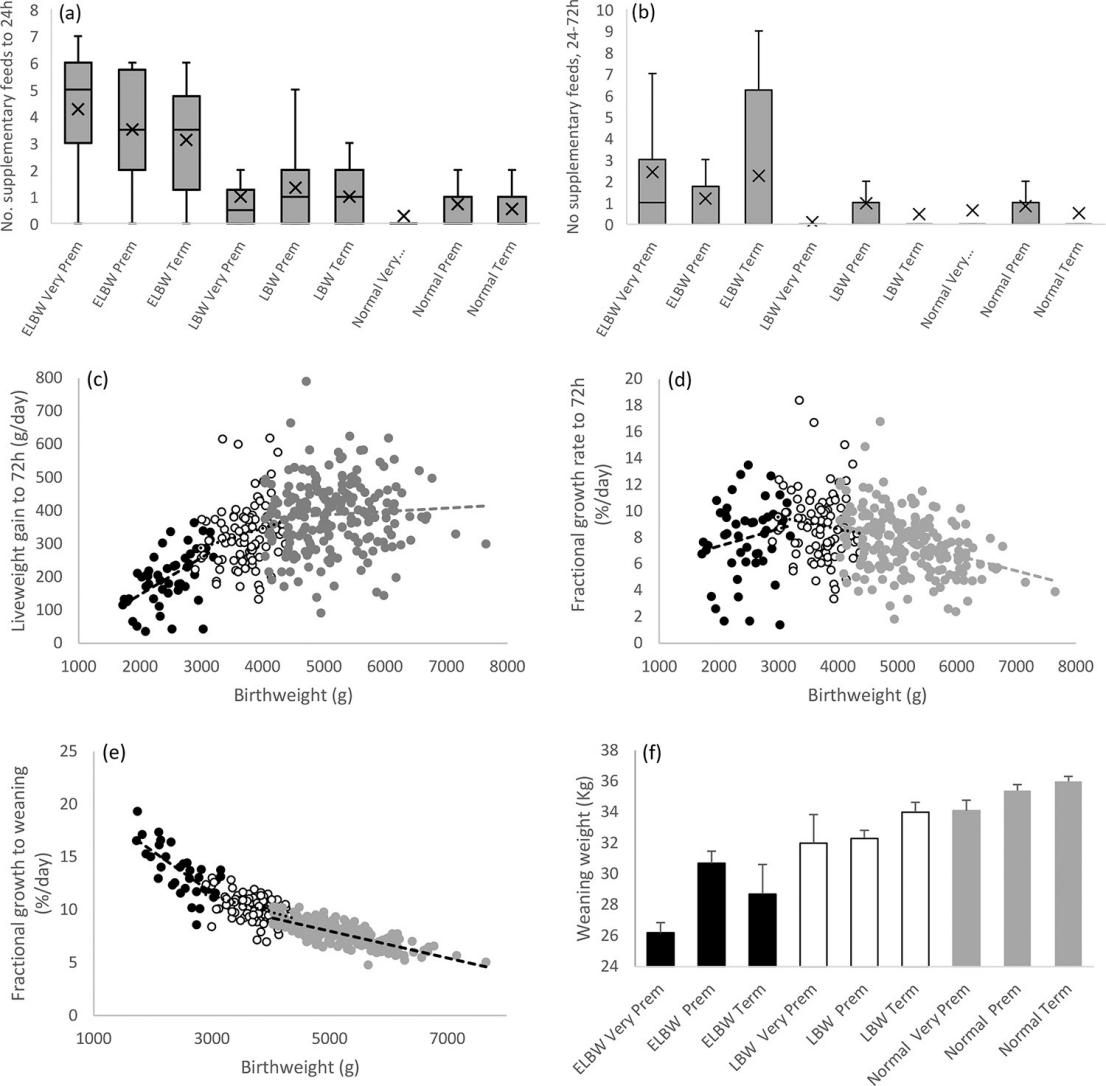
Supplementary feeding and indices of growth in relation to birthweight and delivery categories. Boxplots illustrating the number of supplementary feeds from 4 to 24h (a), and between 24 to 72h (b) after birth in relation to birthweight and delivery classifications, with mean (X) and exclusive median (^_^). Relationship between birthweight and neonatal liveweight gain (c), and between birthweight and fractional growth rate to 72h (d) and 77 days postnatal (e), together with final weight at weaning for birthweight and delivery categories (f). For (a) and (b) birthweight was significant (P<0.001/P = 0.001), but not delivery category (P = 0.362/P = 0.974), or the interaction (P = 0.166/P = 0.173). For extremely low (black circles), low (open circles) and normal (grey circles) birthweight lambs in (c) r = 0.552 (P<0.001), 0.206 and 0.066 (both ns); in (d) r = 0.190, -0.120 (both ns) and -0.357 (P<0.001); in (e) r = -0.709, -0.423 and -0.766 (all P<0.001). In (f) birthweight (P<0.001) and delivery (P = 0.034) categories, and sex (males>females, P<0.001) were significant but none of the interactions between them.

For the study population as a whole, 207 of 350 lambs that survived to weaning at 77days of age were judged to require some degree of supplementary feeding during the first three days postnatal. The number of supplementary feeds required during the first 24h (colostrum), and from 24 to 72h of age (milk) was extremely variable, but highly dependent on birthweight category (P<0.001 and P = 0.001, respectively, Fig 3A and 3B).

Surprisingly, the rate of supplementary feeding was independent of delivery category *per se* (P = 0.362 and 0.974), and there was no birthweight x delivery interaction (P = 0.166 and 0.173). This information provides context for the absolute and fractional growth rate observed during the early neonatal period as a whole. Average absolute growth or liveweight gain (± sem) was 204±12, 331±10 and 388±7 g/day for the ELBW, LBW and normal lambs, respectively (P<0.01 for all *post hoc* comparisons), during the first three days postnatal. In addition, for the population as a whole the expected positive relationship between individual birthweights and liveweight gain was evident (r = 0.494, n = 350, P<0.001), and this was predominantly due to the association within the ELBW lambs, a number of which were struggling to gain weight (Fig 3C, r = 0.505, n = 47, P<0.001). Accordingly, fractional growth rate during the early neonatal period was higher in LBW versus normal lambs (8.9±0.27 and 7.6±0.15%/day, P<0.01 *post hoc*) but intermediate in the ELBW group (8.1±0.41%/day). Thus, while the expected negative relationship between birthweight and fractional growth rate in the early neonatal period was significant at a population level (r = -0.245, P<0.001), this was mainly due to the association within the normal birthweight group (r = -0.357, n = 213, P<0.001, Fig 3D). A much more robust negative association was evident when fractional growth rate was calculated for the first 77 days postnatal, at a population level (r = -0.881, n = 245, P<0.001), and in all three birthweight groups separately (Fig 3E). For the latter, average FGR to weaning was 13.6±0.35, 10.2±0.12 and 7.8±0.07%/day for the ELBW, LBW and Normal groups (P<0.01, *post hoc*). In spite of this markedly higher relative growth rate over the entire period of lactation, extremely low birthweight lambs of both sexes remained lighter at weaning (Fig 3F). Multiple regression was used to further interrogate the relationship between pregnancy outcome parameters, maternal dystocia, initial lamb vigour and feeding interventions on growth to weaning. Maternal dystocia and initial lamb vigour categories were without influence and hence were excluded from the final model. As detailed in Table 7, lamb birthweight and sex were highly predictive of both liveweight gain and fractional growth to weaning. The number of feeding interventions also predicted liveweight gain and thus final weight at weaning but the coefficient was negative indicating that no absolute growth advantage was imparted to the lambs receiving the greatest number of supplementary feeds. When a similar approach was limited to the ELBW group only, lamb sex was the only significant predictor (P = 0.005) of absolute growth rate (data not shown).

**Table 7:** Impact of gestation length, birthweight, sex and supplementary milk feeding in the neonatal period on growth to weaning at 77days of age in singleton lambs (n = 341).

	Liveweight gain to weaning, g/day		Fractional growth to weaning, %/day	
	Β coefficient (SE)	P-value	Β coefficient (SE)	P-value
Gestation length, days	-0.537 (0.986)	0.586	0.037 (0.030)	0.208
Birthweight, kg	16.42 (2.45)	**<0.001**	-1.953 (0.073)	**<0.001**
Colostrum yield, g	-0.017 (0.008)	0.051	-0.0000 (0.0002)	0.858
Female sex	-26.86 (4.06)	**<0.001**	-0.667 (0.122)	**<0.001**
No. of supplementary milk feeds, 4 to 72h	-3.017 (0.758)	**<0.001**	0.036 (0.0228)	0.108

No. of supplementary feeds varies from 0 to 19 (excludes initial colostrum feed).

## Discussion

### Optimal birthweight and gestation length

In the present study the average birthweight of singleton lambs in the optimally-nourished control adolescents (5.3kg) corresponded to that of similarly managed adolescents of equivalent age and size at conception, but differing genotype (5.2kg [29]). It also matched that of singleton-bearing adult ewes of both equivalent (primiparous, 5.2kg [25]) and contrasting genotypes (multiparous 5.5kg, [30]), that were nutritionally managed to meet fetal nutrient requirement in the final third of pregnancy. Accordingly, the majority of control group fetuses were considered to have achieved their prenatal growth potential and to provide a valid point of reference for optimal birthweight. Based on different multipliers of the standard deviation around the mean for the control group, two categories of prenatal growth restriction for the population as a whole were defined, namely extremely low birthweight (<3 SD) and low birthweight (<1.5 SD), and this approach allowed us to compare perinatal complications relative to normally grown lambs. Prior embryo transfer studies have demonstrated that gestation length is influenced by lamb genotype [31] and maternal age [32], and the average difference attributed to these factors is a modest 1.5 and 2 days, respectively. In the present study, the genetic make-up of the embryos (lambs) and the embryo recipients was standardised across all pregnancies, and the average gestation length of controls (145 days) was two days shorter than in adult pregnancies with an identical maternal and fetal genotype (147 days, [25]). Thus, 145 days was considered to represent normal term delivery in this adolescent population and again our approach was to use the standard deviation around this mean to define two categories of preterm delivery. Further, the standard deviation in gestation length of 1.9 days was similar in magnitude to that reported for singleton births in other large breeding flocks [33], and emphasises that the incidence of preterm birth out with this range is a relatively rare phenomenon in otherwise normal ovine pregnancies.

### Preterm delivery, birthweight and the incidence of dystocia

In contrast, spontaneous preterm delivery has been a consistent feature of pregnancy outcome in overnourished adolescent sheep [17, 18, 26, 34], and is robustly supported by the data herein. This large retrospective analysis of more recent studies reveals the distribution of gestation length is shifted to the left with 18.5% of initially viable lambs born between 135 and 139 days of gestation with the majority of these additionally categorised as being of extremely low (52%) or low (24%) birthweight. Spontaneous preterm delivery of a similar magnitude has been reported in a small number of pregnancies following periconception undernutrition but in that case none of the lambs survived even although birthweight *per se* was unperturbed [35, 36]. A number of preclinical studies using sheep models have reported gestation lengths similar to, or shorter than, that occurring naturally here. However, these involve an induced labour in normally growing fetuses, usually following fetal lung maturation with exogenous steroids, and with or without subsequent ventilation once the lamb is delivered [37–39]. While being premature and small is undoubtedly challenging for the lambs themselves (see below) the overnourished mothers had a largely atraumatic delivery and it was the control adolescents that required the greatest degree of obstetrical assistance for fetopelvic disproportion and/or malpresentation. Control-fed adolescents had not reached mature body size by the end of pregnancy [40], but were carrying fetuses that had achieved their prenatal growth potential, thus it is unsurprising that they required more birthing assistance due to a relatively small pelvic size. Others have reported that dystocia is associated with a longer parturition in young first parity ewes [41] but all ewes in the present study were continuously supervised and obstetrical assistance readily provided, curtailing protracted delivery. Consequently, there were no maternal deaths and only one lamb died due to birth injury during a breach delivery. For the population as a whole the degree of obstetrical assistance required to deliver the singleton lambs largely increased with higher birthweight, as widely reported previously [42]. The exception to this relationship was the nine pregnancies where the cervix failed to soften and dilate appropriately (Ringwomb). All cases were in overnourished pregnancies and involved lambs with low or extremely low birthweights. Given that placental mass was attenuated in these pregnancies (average total cotyledon weight 51g), it is probable that the normal cascade of placentally derived steroids that precede parturition, namely a fall in progesterone and rise in oestrogen, was perturbed and/or insufficient leading to impaired cervical softening and myometrial contractility [43, 44]. In support and relative to control-fed adolescents, low circulating progesterone and oestradiol-17β throughout the final third of pregnancy, and an early decrease in peripheral progesterone concentrations are characteristic of overnourished adolescent dams, and closely correlated with placental mass [45, 46].

### Initial colostrum yield, lamb vigour and maternal behaviour

The prenatally growth-restricted lambs largely enjoyed an easy extra uterine passage and initial lamb vigour as measured by the ability to suck the first feed of colostrum from a standard baby bottle was universally high and independent of gestational intake, birthweight and delivery categories. While this may seem like an unusual index of lamb vigour it was necessitated by the requirement to check initial colostrum yield and return that colostrum to the lamb, with additional banked colostrum where needed. Similarly high vigour at birth, based on teat seeking behaviour/level of feeding assistance was reported in lambs from naturally mated singleton bearing adolescents offered equally contrasting pregnancy rations to those reported here [29]. While it might have been anticipated that the ELBW and very premature lambs may have been less vigorous at birth we have recently reported that fetal weight-specific perirenal fat mass, the dominant fat depot and important energy source at birth, is higher in growth-restricted than in normal fetuses at day 130 of gestation [21]. Moreover, the expression of uncoupling protein 1, a key marker of brown adipose tissue functionality was markedly enhanced commensurate with an early preparation for thermogenesis, and indicates that the small lambs were well prepared for *ex-utero* life. Weak lambs were fed the initial colostrum by stomach tube as a last resort. While using a stomach tube to deliver colostrum can be a life-saver in many situations, and particularly when lambing twins and triplets in adverse weather conditions outdoors, we were cognisant that such an approach could impair the natural suckling impulse and decrease the lamb’s motivation to seek the teat subsequently. Indeed, tube-fed lambs are less active and receive less maternal care than their naturally suckled sibling [47], and where routinely practised tube feeding increases the risk of neonatal mortality [48]. Interestingly, in the present study the small number of lambs that were tube-fed had a 12 fold higher risk of mortality after adjusting for gestation length and birthweight. Colostrum supply is fundamental to avoiding starvation, hypothermia and immunocompromise in lambs [1], and when that supply is chronically low (0-50ml) as observed in many of the pregnancies complicated by ELBW and very preterm delivery herein, it takes time for lactation to become established. Moreover, while the vast majority of lambs are initially keen to suckle they rarely persist if the udder is empty. Starvation was avoided by our supplementary feeding/suckling assistance strategy and unsurprisingly it was the ELBW lambs that were more likely to require assistance during the first 24h of life, and throughout the neonatal period. Similarly, and in part as a consequence of this essential intervention to mitigate against inadequate colostrum and/or milk supply, ELBW lambs were also at highest risk of experiencing an impaired ewe-lamb bond during the neonatal period. In six cases overnourished dams harmed the lamb to such an extent that it died or had to be put down. This extreme behaviour could relate to rapid unassisted delivery, low birthweight, premature delivery and inadequate colostrum supply, all of which were a feature of the individual pregnancies concerned. Add to this the fact that the mothers had no prior experience leads to a perfect storm for poor bonding. Indeed both primiparous and adolescent mothers display a range of poor mothering behaviours including inappropriate movements when the lamb attempts to suckle, a latency to reunite when separated, and in extreme cases aggression [49, 50], all of which make the lamb vulnerable to physical harm, particularly when housed in an individual pen.

### Neonatal morbidity and mortality

It is well established that low birthweight lambs are more susceptible to neonatal morbidity and mortality (see introduction) but the strength of the current data set is that it additionally allows us to examine the impact of temporal prematurity. The most prevalent morbidity issues related to establishing appropriate respiration at birth (incidence 8.6%) and renal complications subsequently (5.3%), and the risks of both were greatest in ELBW lambs. Although these growth-restricted lambs are mildly hypoxic and modestly hypoglycaemic compared with normally growing controls in late gestation [51], lung mass is appropriate for fetal size [14]. Thus, the initial transient respiratory issues most likely relate to differences in the maturation of the surfactant system as previously reported in growth-restricted fetuses from the carunclectomy model at day 133 and 141 of gestation [52]. We were not in a position to measure accepted indices of kidney function directly in lambs with suspected renal issues but the observed low urine volume and presence of blood or protein in urine samples when obtained was commensurate with observations in growth-restricted human neonates and most likely reflected a similar deficit in nephron number [53]. In support and in this paradigm, total nephron number is correlated with fetal weight in late gestation (r = 0.540, n = 47, P<0.001), and reduced in prenatally growth restricted (n = 22) versus normal birthweight offspring (n = 20) in adulthood (119±5.2 x 10^4^ versus 159±6.7 x 10^4^, P<0.001, JM Wallace and JS Milne unpublished data). Thus, potentially overloading the neonatal kidney by ensuring the lambs had adequate colostrum was likely the route problem. Irrespective our remedial strategy to offer lambs with suspected renal issues a short period of electrolyte fluid replacement alleviated the problem and allowed appropriate renal function to resume. This is in line with a previous report indicating that electrolyte therapy in sick lambs was highly protective against perinatal mortality [54].

It was very preterm delivery rather than low birthweight that was primarily associated with an increased risk of gastrointestinal tract issues, namely persistent diarrhoea, requiring additional antibiotic therapy (incidence 3.5%). Relative to fetal body weight the ovine gastrointestinal tract grows rapidly in late gestation [55] and undergoes sweeping ontogenic changes in preparation for appropriate digestion and absorption of nutrients, bioactive compounds, immunoglobulins, cytokines and immune cells from colostrum [56]. It is axiomatic that when the lamb is delivered early the gut is unlikely to be fully mature leaving the intestinal epithelium vulnerable to colonization by the harmful pathogens commonplace in indoor lambing systems. There is a paucity of supporting data in spontaneously preterm lambs but the density of goblet cells which are primary involved in forming an appropriate protective mucosal layer to restrict such microbial colonisation is lower in preterm piglets at birth and at term equivalent age [57]. Further, calves born on average 4 days early in heat-stressed versus cooled dairy cows have lower efficiency of IgG absorption and hence reduced passive immunity irrespective of colostrum source [58]. The latter colostrum cross-over study demonstrates that it is the immaturity of the neonatal gut rather than the quality of the colostrum that is the main issue. We have measured colostrum IgG concentration in the majority, but not all, of the pregnancies studied here and this was independent of delivery category (107.5, 91.1 and 93.8 mg IgG/ml in the very premature, premature and terms groups, respectively, P = 0.222, JM Wallace and JS Milne unpublished data). Irrespective, the low colostrum volume necessitated that many of the very premature lambs additionally received banked colostrum and IgG absorption *per se* was not routinely assessed. It is accepted that the use of prophylactic antibiotics could negatively impact the neonatal microbiota but all lambs were treated identically and our approach to neonatal care was justified by the complete absence of E.coli enterotoxaemia (watery mouth), umbilical infection (navel ill) and infectious polyarthritis (joint ill), all of which are commonplace on farm [59, 60].

In spite of the above neonatal complications all-cause mortality for the population as a whole was exceptionally low (5.4%) and reflected a mix of parturition-related and neonatal deaths including the lambs euthanased following maternally-induced injuries. It is our firm belief that the proactive and intensive neonatal care regime involving the ready availability of obstetrical assistance, good hygiene, prophylactic antibiotics, extensive monitoring of well-being and appropriate nutritional supplementation effectively mitigated against a higher number of neonatal deaths. Given that low birthweight is a widely accepted predictor of lamb mortality [1, 3, 4] it was unsurprisingly to find that the ELBW lambs had a 7 fold higher mortality risk than normal birthweight lambs. In contrast, there are few reports of mortality after spontaneous delivery at the early gestational age recorded here but the limited evidence indicates that the majority of lambs did not survive (100% mortality after delivery at 139 days, [35, 36]; 25 and 90% mortality at 140–143 days and 137–139 days, respectively [12]). It is remarkable therefore that in the present study 80% of the 50 lambs born between 135 and 139 days gestation survived to weaning, while survival in those born between 140 and 143 days gestation was 97%.

### Growth to weaning

While our intensive approach to neonatal care involving widespread nutritional supplementation was considered essential for maximising lamb survival it could in theory confer a relative growth stimulus compared with unsupplemented lambs. This is an important consideration given that lambs from individual years were often part of longitudinal fetal programming studies. Nonetheless in spite of a high degree of supplementation it was individual ELBW lambs that initially struggled to gain weight in the first 3 days after birth, and for the population as a whole absolute growth to weaning was negatively predicted by the number of supplementary feeds suggesting that no overall growth advantage ensued. This is in line with a previous study which showed that neonatal supplementary feeding to ensure survival did not impact key indices of growth, glucose metabolism or body composition at weaning [61]. Moreover it was birthweight that predicted growth to weaning in the present analysis and in spite of rapid fractional growth during the suckling period, the ELBW lambs remained lighter at weaning. This aligns with observations of persistently reduced stature and bone mineral density in adult life in both prenatally-growth restricted lambs [62], and prematurely delivered ELBW humans [63], and contrasts with the early and complete compensatory catch-up growth reported in the ovine carunclectomy model [64].

### Sex effects

Previous studies have highlighted that male lambs are more likely to experience birth difficulty, low vigour, and neonatal mortality than females [1, 8, 37, 65]. However in the present study we found no evidence of an imbalance in the sex ratio within very premature or extremely low birthweight categories. Males were heavier than females and hence sex-specific cut-offs were used to define birthweight categories but irrespective we found no evidence of male bias in the requirement for assistance at delivery or in initial lamb vigour. Similarly sex did not influence the risk of morbidity or mortality, and as expected males grew faster than females from birth to weaning reflecting their greater anabolic drive to lean tissue growth at this life stage [61, 66].

## Conclusion

In summary, premature delivery, low birthweight and inadequate colostrum supply are commonplace when still-growing adolescent dams are overnourished to promote their own body growth. Although the offspring have a relatively easy extra uterine passage and are initially vigorous, the subsequent neonatal period is hazardous and the smallest and most premature lambs are most vulnerable to mismothering, starvation, and a number of associated perinatal complications. The proactive approach to neonatal care described here is undoubtedly labour intensive but it demonstrates that most of the issues associated with spontaneous preterm delivery and low birthweight can be overcome with the vast majority of young adolescent ewes successfully rearing a lamb to weaning. While our goal within a research environment was to ensure survival as part of longitudinal fetal programming studies there are simple lessons which may improve lamb survival in indoor lambing systems on farm. Of these continuous supervision during the expected delivery period, frequent monitoring of lamb wellbeing by observation and weight recording, and ensuring adequate colostrum throughout the first 24h postnatal are considered fundamental.

## Supporting information

S1 File Individual animal data. (XLSX)
